# Transcriptional and posttranscriptional regulation of *Shigella shuT* in response to host‐associated iron availability and temperature

**DOI:** 10.1002/mbo3.442

**Published:** 2017-01-26

**Authors:** Yahan Wei, Andrew B. Kouse, Erin R. Murphy

**Affiliations:** ^1^ Department of Biological SciencesOhio UniversityAthensOHUSA; ^2^ Cell Biology and Metabolism ProgramNICHDNIHBethesdaMDUSA; ^3^ Department of Biomedical SciencesOhio University Heritage College of Osteopathic MedicineAthensOHUSA

**Keywords:** Fur, gene regulation, heme‐uptake, regulatory mechanisms, RNA thermometer, *Shigella dysenteriae*

## Abstract

Like most bacteria, *Shigella* must maintain a precise balance between the necessity and toxicity of iron; a balance that is achieved, at least in part, by regulating the production of bacterial iron acquisition systems in response to specific environmental signals. Using the *Shigella* heme utilization (Shu) system, *S. dysenteriae* is able to acquire iron from heme, a potentially rich source of nutritional iron within the otherwise iron‐limited environment of the human host. Investigations presented within reveal two distinct molecular mechanisms underlying previously uncharacterized transcriptional and translational regulation of *shuT*, a gene encoding the periplasmic‐binding component of the Shu system. While *shuT* transcription is regulated in response to iron availability via a process dependent upon the global regulator Fur and a Fur‐binding site located immediately downstream of the promoter, *shuT* translation is regulated in response to environmental temperature via the activity of an RNA thermometer located within the 5′ untranslated region of the gene. Such complex regulation likely increases the fitness of *S. dysenteriae* by ensuring maximal ShuT production when the pathogen is within the iron‐limited and relatively warm environment of the infected host, the only environment in which heme will be encountered as a potential source of essential iron.

## 1 INTRODUCTION

*Shigella* is a genus of pathogenic enterobacteria composed of four species, *S. boydii*,* S. sonnei*,* S. flexneri*, and *S. dysenteriae. Shigella* species are the causative agents of shigellosis, a form of severe infectious bacillary dysentery in humans, with a very low infectious dose (~10–100 cells) (DuPont, Levine, Hornick, & Formal, 1989). It is estimated that there are no <164 million infections and 1.1 million deaths caused by shigellosis annually, with the majority of both infections and deaths occurring in children under the age of five (Kotloff et al., 1999). Of the four *Shigella* species, *S. dysenteriae* is the only one known to cause epidemic outbreaks, has the highest fatality rate following infection, and is associated with significant postinfection sequelae including Hemolytic Uremic Syndrome and Reiter's syndrome (Calin, 1979; Kotloff et al., 1999; Mark Taylor, 2008). These facts in combination with the lack of a vaccine to prevent shigellosis and no universally safe antibiotic regimen to treat the infection makes understanding the molecular mechanisms underlying the infection with *S. dysenteriae* of utmost importance. It is only after such an understanding is achieved that this information can be used to guide the development of novel therapeutics designed to disrupt specific virulence‐associated processes, and by doing so, lessen or eliminate the ability of *Shigella* to cause human disease.

Following entry into the host via the fecal‐oral route, a successful *Shigella* infection requires sequential invasion into colonic epithelial cells, intracellular replication, and cell‐to‐cell spread by the pathogen. Collectively, these processes disrupt water balance within the colon and promote the formation of lesions within the colonic epithelium; processes that result directly in the bloody diarrhea associated with *Shigella* infections (Schroeder & Hilbi, 2008). During each stage of the infection process, both within the host and during transmission, *Shigella* must acquire iron. Like that of most bacterial species, iron is essential to survival of *Shigella* due to its critical role as a cofactor to enzymes involved in such processes as respiration and DNA replication. In the nonhost environment, iron is relatively abundant and bio‐available to *Shigella* and other bacteria. In the human body, however, as a result of host iron utilization and an innate host immune defense against infection, the level of bio‐available iron is maintained at exceedingly low concentrations. One mechanism by which low free iron levels are maintained within the human host is be sequestration of the element within host compounds, such as heme. Binding approximately 95% of all iron within the human body, heme represents the most abundant potential source of nutritional iron for an invading pathogen if the pathogen is able to utilize the iron bound within (Otto, Verweij‐van Vught, & MacLaren, 1992). The utilization of heme‐bound iron is one mechanism by which some pathogenic bacteria overcome the extreme iron limitation encountered within the host. Examples of heme utilization systems in pathogenic bacteria include, but are not limited to, the Chu system present in some strains of pathogenic *Escherichia coli*, the Has system in *Serratia marcescens*, the Bhu system in *Bordetella* species, the Hut system in *Bartonella quintana*, and multiple heme acquisition systems in *Haemophilus influenzae* (Ghigo, Letoffe, & Wandersman, 1997; Murphy et al., 2002; Parrow, Abbott, Lockwood, Battisti, & Minnick, 2009; Vanderpool & Armstrong, 2001; Whitby, Seale, VanWagoner, Morton, & Stull, 2009; Wyckoff et al., 1998). Importantly, the ability of a bacterial pathogen to utilize heme as a source of nutritional iron is often positively associated with virulence (Otto et al., 1992).

The ability of *S. dysenteriae* to utilize heme as an iron source is strictly dependent upon the activity of the *Shigella* heme uptake (Shu) system (Mills & Payne, 1995; Wyckoff et al., 1998). Components of the Shu system are encoded within the *shu* locus, a 9.1 kb region predicted to contain eight genes under the control of four predicted promoters. The predicted arrangement of genes and promoters within the *shu* locus would result in the generation of two monocistronic transcripts (*shuA* and *shuS*), and two polycistronic transcripts (*shuTWXY* and *shuUV*) (Wyckoff et al., 1998). The essential roles that Shu proteins play in the ability of *S. dysenteriae* to utilize heme as a sole source of iron has been demonstrated in multiple studies (Burkhard & Wilks, 2007, 2008; Eakanunkul et al., 2005; Wyckoff, Lopreato, Tipton, & Payne, 2005; Wyckoff et al., 1998). Despite a well‐established role in the essential process of iron acquisition, direct measurement of the contribution of the Shu system to *S. dysenteriae* pathogenesis is limited given the lack of availability to relevant in vivo infection models and the limitations of in vitro tissue culture‐based virulence assays. However, studies have indicated a positive association between the Shu or orthologous systems and virulence (Mills & Payne, 1995; Torres, Redford, Welch, & Payne, 2001; Wyckoff et al., 1998). Specifically, inactivation of *chuA*, the *shuA* ortholog in uropathogenic *E. coli*, diminishes the ability of the strain to colonize mice as compared to that of the wild‐type strain (Torres et al., 2001). Additionally, the fact that only pathogenic bacterial strains encode the *shu* or orthologous genes, such as *chu* genes in pathogenic *Escherichia coli* and *hmu* genes in *Yersinia* species, support the conclusion that the ability to utilize heme as a source of nutritional iron facilitates bacterial virulence including, most likely, that of *Shigella* species (Mills & Payne, 1995; Wyckoff et al., 1998).

While iron is essential, the element is toxic when in excess (Everse & Hsia, 1997). As a means to maintain the critical balance between the necessity and toxicity of iron, the production of bacterial iron‐uptake systems is tightly regulated. One such regulatory mechanism utilized by a wide variety of bacteria is that of transcriptional modulation by Fur, an iron‐responsive transcriptional regulator (Bagg & Neilands, 1987). In these cases, Fur functions to repress transcription by binding in an iron‐dependent manner to specific sequences within the promoter region of each target gene (Troxell & Hassan, 2013). Moreover, as potential sources of nutritional iron vary depending upon the environment, the production of different iron acquisition systems by pathogenic bacteria is often regulated in response to multiple environment‐specific signals. In addition to iron availability, the production of specific bacterial iron acquisition systems has been shown to be influenced by such environmental signals as oxygen levels and temperature (Battisti, Sappington, Smitherman, Parrow, & Minnick, 2006; Carpenter & Payne, 2014; Kouse, Righetti, Kortmann, Narberhaus, & Murphy, 2013; Vanderpool & Armstrong, 2003; Wei & Murphy, 2016a). Such multi‐factored regulation likely ensures the production of a given iron acquisition system when, and only when, a bacterium is within an environment likely to contain the corresponding iron source.

Production of *S. dysenteriae* ShuA, the outer‐membrane heme receptor of the Shu system, is known to be regulated in response to multiple environmental‐specific signals. Specifically, *shuA* has been demonstrated to be subject not only to iron‐dependent transcriptional regulation mediated by Fur, but also to temperature‐dependent posttranscriptional regulation mediated by an RNA thermometer located within the 5′ untranslated region (5′UTR) of the gene (Kouse et al., 2013; Mills & Payne, 1997). RNA thermometers are *cis*‐encoded ribo‐regulators that inhibit translation of the target gene in which they are housed. At nonpermissive temperatures, an inhibitory hairpin is formed within the regulated transcript that physically occludes the Shine‐Dalgarno (SD) sequence, thus preventing ribosomal binding and translation initiation (Kortmann & Narberhaus, 2012). RNA thermometers are most often implicated in regulating the production of heat shock proteins and specific bacterial virulence determinants (Kortmann & Narberhaus, 2012; Wei & Murphy, 2016b). The characterization of RNA thermometer‐mediated regulation of *S. dysenteriae shuA* was the first demonstration of such regulation affecting the expression of a gene required for nutrient acquisition (Kouse et al., 2013).

Aside from *shuA,* little is known about the expression of any other gene within the *S. dysenteriae shu* locus. This study aims to identify and characterize the regulatory mechanism(s) controlling the expression of *shuT,* a gene encoding the periplasmic heme‐binding component of the Shu system. The findings of this study provide the first direct evidence that *shuT* expression is regulated in response to two different host‐associated environmental cues, and elucidates the molecular mechanism governing each. Specifically, it is demonstrated that *shuT* is subject to: (1) iron‐dependent transcriptional regulation that is mediated by Fur and dependent upon a Fur‐binding site located immediately downstream of the transcriptional start site of the gene, and (2) temperature‐dependent posttranscriptional regulation mediated by an RNA thermometer located within the *shuT* 5′ UTR. Such multilevel regulation likely increases the fitness of *S. dysenteriae* by ensuring that ShuT production is limited to times within the infection cycle when the pathogen is within the human host, the only environment where it will encounter heme as a potential source of essential iron.

## 2 EXPERIMENTAL PROCEDURES

### 2.1 Strains and culture conditions

All bacterial strains and plasmids used in this research are shown in Table S1. *Escherichia coli* was cultured in Luria‐Bertani (LB) broth (1% tryptone, 0.5% yeast extract, and 1% NaCl) or on LB agar plates (LB with 1.6% [w/v]) at 37°C. *S. dysenteriae* was cultured in LB broth or on Tryptic soy broth agar plates (Becton, Dickenson and Company, Sparks, MD) containing 0.01% (w/v) Congo red dye (ISC BioExpress, Kaysville, UT) at the indicated temperatures. “Iron‐rich” media (+Fe) refers to the LB broth, whereas “iron‐poor” media (−Fe) refers to LB broth containing 150 μmol L^−1^ 2,2′‐bipyridine (Alfa Aesar, A Johnson Matthey Company) as an iron chelating reagent. Chloramphenicol and Ampicillin were used at a final concentration of 30 (μg ml^−1^) and 150 (μg ml^−1^), respectively, for the growth of bacterial strains carrying plasmids. Specifically for the heme utilization assay, 2,2′‐bipyridine with a final concentration of 225 μ mol L^−1^ was added to LB broth to chelate iron from the culturing media, generating “iron‐poor” conditions, under which about 90% of wild type *S. dysenteriae* growth is inhibited. To generate the growth condition in which heme represents the major source of iron (heme), heme (Sigma) was added to the –Fe media at a final concentration of 100 (μg ml^−1^), a minimal concentration determined to permit maximal recovery of wild‐type *S. dysenteriae* growth.

### 2.2 Oligonucleotide primers

All oligonucleotide primers were designed based on the chromosomal sequences of *S. dysenteriae* and synthesized by Integrated DNA Technologies. Primer sequences used in this research are summarized in Table S2.

### 2.3 Reporter plasmid construction

pT*‐lacZ*: Complementary oligonucleotides containing the nucleic acid sequences of the putative *shuT* promoter region (Table S2) were hydrated in STE buffer (0.1 mol L^−1^ NaCl, 10 mmol L^−1^ Tris‐HCl, 1 mmol L^−1^ EDTA, pH 8.0). The putative *shuT* promoter region was reconstructed by combining the complementarity oligonucleotides, boiling the molecules in a water‐bath for 10 min and allowing them to cool slowly to room temperature. The annealed double‐stranded DNA molecule was then ligated into plasmid pHJW20 (Castellanos et al., 2009) digested with restriction enzymes SalI (New England Biolabs inc.) and XbaI (New England Biolabs Inc.). Such cloning placed the DNA fragment containing the putative *shuT* promoter region immediately upstream of the report *lacZ* gene of pHJW20.

pT‐UTR: Primers were designed to amplify the region containing the promoter and full 5′‐UTR of *shuT* (Table S2) by PCR using genomic DNA of wild‐type *S. dysenteriae* as template. The amplified fragment was purified by gel extraction and digested with restriction enzymes AatII (New England Biolabs Inc.) and NheI (New England Biolabs Inc.). The digested fragment was then purified and ligated into plasmid pXG10 (Urban & Vogel, 2007) that had been digested with AatII (New England Biolabs Inc.) and NheI (New England Biolabs Inc.) to generate plasmid pT‐UTR. Such cloning removed the PLtetO‐1 constitutive promoter of pXG‐10 and placed the promoter and full 5′ UTR of *shuT* immediately upstream of the reporter *gfp* gene.

pF‐UTR, pS‐UTR, and pD‐UTR: Complementary oligonucleotide primers containing the indicated nucleic acid sequences (Table S2) were combined and annealed in STE buffer (0.1 mol L^−1^ NaCl, 10 mmol L^−1^ Tris‐HCl, 1 mmol L^−1^ EDTA, pH 8.0) by boiling in a water‐bath for 10 min followed by slow cooling to room temperature. The generated double‐stranded DNA products were then ligated into plasmid pXG10 (Urban & Vogel, 2007) that had been digested with restriction enzymes NsiI (New England Biolabs Inc.) and NheI (New England Biolabs Inc.). Such cloning placed the DNA fragment encoding the putative RNA thermometer being investigated between the PLtetO‐1 constitutive promoter and the reporter *gfp* gene of pXG‐10.

### 2.4 DNA sequencing

All DNA sequencing was completed at the Ohio University Genomics Facility using Big Dye chemistry and analyzed on an Applied Biosystems 3130xL Genetic Analyzer.

### 2.5 RNA extraction and DNA removal

Total RNA was harvested from wild‐type *S. dysenteriae* cultured to the midlogarithmic phase under the indicated growth condition. Following growth to midlogarithmic phase, RNA preserving buffer (95% ethanol and 5% phenol, pH 4.5) was added to each culture at a culture:buffer ratio of 4:1, and the mixture incubated at 4°C overnight. Following overnight incubation, cells present in 3 ml of bacterial culture were pelleted by centrifugation at 17,000 ***g*** for 2 min. After discarding the supernatant, bacterial cells were resuspended in 357.3 μl DEPC‐treated ddH_2_O, 40 μl 10% (w/v) sodium dodecyl sulfate (SDS), and 2.67 μl 3 mol L^−1^ sodium acetate (pH 5.2) by vortexing for 15 s. Lysis of the bacterial cells was achieved by incubating each resuspended sample at 90°C for 7 min. After lysis, 1 ml of Trizol reagent (Ambion) was added to each sample, the sample transferred to a 2 ml phase‐lock gel tube (5 PRIME Inc., Gaitherburg, MD) and the reaction incubated at room temperature (~25°C) for 5 min. Trizol reagent was extracted from each sample by the addition of 250 μl chloroform followed by vigorous shaking for 30 s to 1 min and then incubation at room temperature for 2 min. Each sample was then subjected to centrifugation for 2 min at 17,000 ***g*** and the nucleic acid containing aqueous phase transferred to a clean 2 ml microfuge tube. One milliliter of 100% ethanol was added to each sample and the samples incubated overnight at −80°C. Following overnight incubation at −80°C, nucleic acid present in each sample was pelleted by centrifugation at 17,000 ***g*** for 15 min at 4°C. Each pellet was washed by the addition of 1 ml cold 70% ethanol followed by centrifugation as described above. The supernatant was then removed and each pellet dried by centrifugation in a vacufuge (Eppendorf) for approximately 2 min in Alcohol Mode. Finally, each RNA pellet was resuspended in 53 μl of nuclease‐free water.

Following DNA removal from each RNA sample using TURBO DNA‐*free* kit (Ambion) as directed, the absence of contaminating DNA was confirmed by PCR analysis; using the RNA sample as template, the lack of amplification by a known primer set indicates the absence of DNA in the RNA sample. Finally, the concentration of the total RNA present in each sample was measured using a ND‐1000 spectrophotometer (NanoDrop Technologies, Wilmington, DE).

### 2.6 Reverse transcriptase PCR

After harvesting total RNA from wild‐type *S. dysenteriae* cultured under the indicated conditions via the procedure dictated above, a cDNA library was generated by using the iScript cDNA Synthesis Kit (Bio‐Rad) as directed. PCR amplification was conducted to identify the approximate *shuT* transcriptional start site by using the generated cDNA library as template. Sequences of the four forward primers (shuT‐F1, shuT‐F2, shuT‐F3, and shuT‐F4) and one conserved reverse downstream primer (shuT‐R) are detailed in Table S2. Validation of each primer set was achieved by using each in a PCR reaction containing genomic DNA of wild‐type *S. dysenteriae* as template. Total RNA was used as PCR template with each primer set to confirm the absence of contaminating DNA; and double‐distilled sterilized water was used as PCR template to confirm the absence of DNA contamination in reagents used for the PCR reaction. PCR products were detected by gel electrophoresis and UV light detection.

### 2.7 5′‐RACE analysis

Following isolation of total RNA from *S. dysenteriae* cultured to the midlog phase under iron‐poor conditions at 37°C and DNA removal (as detailed above), ribosomal RNA was depleted from each sample using the RiboMinus Transcriptome Isolation Kit for bacteria (Ambion) as directed, and the RNA sample concentrated by ethanol precipitation. 5′ RACE analysis was completed using the FirstChoice RLM‐RACE Kit (Ambion) with the following modifications. First, approximately 250 ng of mRNAs was treated with the Tobacco Acid Pyrophosphatase provided by the FirstChoice RLM‐RACE Kit prior to adaptor ligation. Secondly, a *shuT*‐specific primer and SuperScript III Reverse Transcriptase (Invitrogen) were used to generate *shuT‐*specific cDNA separately from adaptor‐ligated RNA (experiment group) and RNA without adaptors (control group), which were then used as the templates for PCR amplification. The first round of PCR amplification was performed using the outer primer pair that binds within *shuT* and to the 5′ region of the adaptor sequence (Table S2). Amplified products that were present in the experiment group and absent in the control group were gel purified and used as the templates for a second round of PCR. The second PCR round was completed to increase the specific amplification of *shuT* using the inner primer pair: an upstream primer that binds to *shuT* at a location upstream to that bound by the primer used in the first amplification and a downstream primer that binds to the 3′ region of the adaptor sequence (Table S2). Lastly, the amplified products of the second round of PCR were purified by gel extraction and ligated directly into the plasmid pGEM‐T Easy (PROMEGA, Madison, MI) for nucleic acid sequencing.

### 2.8 Beta‐galactosidase assay

The Miller method was used to complete all beta‐galactosidase analyses (Ausubel et al., 1995; Miller, 1992). Briefly, *S. dysenteriae* containing a given reporter plasmid (pT‐*lacZ*) or pMic21 control vector was cultured at 37°C to stationary phase in 3 ml LB broth and then subcultured in 3 ml LB broth to the logarithmic phase (OD_600_: 0.28–0.7) at 37°C. 30 (μg ml^−1^) Chloramphenicol was added to each culture to ensure maintenance of the reporter or control plasmid. Following incubation of each culture on ice for 20 min, bacteria present in 1 ml of the culture were pelleted by centrifuging at 5,000***g*** for 5 min. Next, each bacterial cell pellet was resuspended in 1 ml Z buffer (0.06 mol L^−1^ Na_2_HPO_4_, 0.04 mol L^−1^ NaH_2_PO_4_, 0.01 mol L^−1^ KCl, 0.001 mol L^−1^ MgSO_4_, 0.05 mol L^−1^ β‐mercaptoethanol, pH 7.0) and the OD_600_ measured using a ND‐1000 spectrophotometer (NanoDrop Technologies, Wilmington, DE). Next, 400 μl of the resuspended cells was diluted 1:1 with Z buffer and the bacterial cells permeabilized by the addition of 50 μl 0.1% SDS and 100 μl chloroform prior to vortexing for 10 s. Following incubation at 30°C for 15 min, 160 μl of 4 (mg ml^−1^), Ortho‐Nitrophenyl‐β‐galactopyranoside (ONPG) was added to each sample and the reaction incubated at 37°C until the sample developed a visible yellow color. Once a given sample developed a detectable yellow color, the time of incubation was noted, 400 μl of 1 mol L^−1^ Na_2_CO_3_ was added to stop the reaction and the sample was subjected to centrifugation at maximum speed for 2 min. The supernatant of each sample was then collected and the OD_420_ and OD_550_ measured using the ND‐1000 spectrophotometer (NanoDrop Technologies, Wilmington, DE). Miller units were calculated using the following equation: $\text{Units}\;\beta -\text{galactosidase}=\frac{1000[{\mathrm{OD}}_{420}-1.75\left({\mathrm{OD}}_{550}\right)]}{t\times V\times {\mathrm{OD}}_{600}}$ (*t* stands for the reaction time in minutes; *V* represents the volume of culture used in milliliters, which, in this case, is 0.4 ml). A reaction containing all reaction components except the cell culture was used as a negative control in each beta‐galactosidase assay. Additionally, *S. dysenteriae* carrying plasmid pMic21, a reporter plasmid containing no cloned promoter, was used to measure background beta‐galactosidase in the strain.

### 2.9 Western blot analysis

All western blot analyses were completed using whole cell extracts. Specifically, *S. dysenteriae* containing the indicated plasmid was cultured to midlogarithmic phase under the indicated conditions and the OD_600_ measured using the ND‐1000 spectrophotometer (NanoDrop Technologies, Wilmington, DE). A total of 5 × 10^8^ bacterial cells were pelleted by centrifugation at 17,000***g*** for 2 min and suspended in 200 μl Laemmli protein dye (Bio‐Rad) containing 5% β‐mercaptoethanol. Samples were boiled for 10 min and then stored at −20°C until use.

Proteins present in 15 μl of each sample were separated on a 7.5% gel using sodium dodecyl sulfate polyacrylamide gel electrophoresis (SDS‐PAGE). PVDF was cut to size, presoaked in methanol for 10 min and rinsed with water prior to transfer of all protein from the acrylamide gel to PVDF membrane at 350 milliamperes for 1 hr. Next, the membrane was blocked in 10% nonfat milk dissolved in PBS with 0.1% (wt ml^−1^) Tween 20 (PBST) overnight at 4°C. After blocking, the membrane was incubated for 1 hr at 4°C in a solution of mouse anti‐GFP antibody (Roche) diluted 1:1000 in 5% milk with PBST. Next, the membrane was washed three times for 15 min each in PBST and blocked in 10% nonfat milk dissolved in PBS with 0.1% (wt ml^−1^) Tween 20 (PBST) prior to incubation for 1 hr at 4°C in a solution of goat anti‐mouse HRP conjugated IgG (Bio‐Rad) diluted 1:20,000 in 5% (wt ml^−1^) milk with PBST. Following incubation with the secondary antibody the membrane was washed with PBST three times for 15 min as detailed above. Then the Chemiluminescent HRP Substrate (Millipore Corporation, Billerica, MA) was added to the membrane, the reaction incubated at room temperature for approximately 3 min and the membrane imaged using the Molecular Imager ChemiDoc XRS+ imaging system (Bio‐Rad). Total protein present on each membrane was visualized by staining with Ponceau S, following completion of the Western blot procedure to ensure even loading of all lanes.

### 2.10 Quantitative real‐time PCR analysis

For samples to be analyzed by both Western blot and quantitative real‐time PCR (qRT‐PCR), total RNAs was harvested from the same culture used to generate the corresponding whole cell protein preparations using the procedures detailed above. After the isolation of total RNA and subsequent DNA removal as detailed above, the iScript cDNA Synthesis Kit (Bio‐Rad) was used to generate a cDNA library as directed. Each cDNA sample was diluted 1:10 in double‐distilled water. Five microliter of diluted cDNA sample was mixed with 10 μl of iTaq Universal SYBR Green Supermix (Bio‐Rad) and 5 μl of each primer set at an optimum concentration, making an amplification reaction mixture with a total volume of 20 μl. All amplification reactions were performed in a CFX96 Real‐Time System (Bio‐Rad) under reaction conditions optimized for each primer set. For each target gene, a six‐point standard curve was generated to ensure that acceptable amplification efficiency was achieved and that all experimental samples amplify within the linear portion of the standard curve. Using the ΔΔCt calculation method the expression level of each target gene was normalized to that of *rrsA* present in each sample and expressed relative to that within a selected control sample. All primers used in qRT‐PCR were designed using Beacon Designer 7.5 and are detailed in Table S2.

### 2.11 Electrophoretic mobility shift assay

#### 2.11.1 Generation of labeled DNA species

Primer pXG10‐for was labeled with ^32^P at the 5′ end by T4 polynucleotide kinase (New England Biolabs Inc.) based on the factory protocol. The radio‐labeled primer was then used to generate ^32^P end‐labeled DNA fragments for EMSA via PCR amplification (Table S2). Plasmid pF‐UTR was used as template in a PCR reaction to generate a DNA fragment containing the putative Fur‐binding site. To generate a nonspecific DNA control fragment, plasmid pXG‐1 was constructed. Specifically, Plasmid pXG10 (Urban & Vogel, 2007) was digested with restriction enzymes NsiI and NheI (New England Biolabs Inc.) in order to remove the DNA sequence between the PLtetO‐1 constitutive promoter and the *gfp* coding region. Next, the digested plasmid was blunted with DNA Polymerase I, Large (Klenow) Fragment (New England Biolabs Inc.), circularized using DNA ligation, and introduced into the host bacterium by transformation. Colonies containing the constructed plasmid were selected for by growth on 30 (μg ml^−1^) Chloramphenicol, and its presence verified by PCR‐based screening. Once constructed, pXG‐1 was purified and used as template to amplify a DNA fragment identical to that generated above but lacking all *shuT‐*derived sequences.

#### 2.11.2 Production and purification of *S. dysenteriae*Fur

PCR primers were designed based on *S. dysenteriae fur* gene sequence to generate a DNA fragment that contains a BamHI and EcoRI recognition site at the 5′ and 3′ end, respectively (Table S2). Purchased plasmid vector pGEX‐2T (GE Healthcare) and the amplified DNA fragment were both digested with BamHI and EcoRI (New England Biolabs) and ligated together to construct plasmid pGEX‐*fur,* the sequence of which was confirmed by nucleic acid sequencing. The constructed plasmid has the *Shigella fur* gene fused in frame with the gene encoding glutathione S‐transferase (GST), all under control of the Ptac promoter. Expression of the GST‐Fur fused protein was induced by 1 mmol L^−1^ isopropyl‐β D‐thiogalactoside (IPTG). After purification with a Sephorase 4B gel (GE Healthcare) column, thrombin (GE Healthcare) was used to cleave the GST tag from the purified GST‐Fur protein. Previously published data has indicated the proper DNA‐binding activity of Fur proteins expressed via this method (Pohl et al., 2003). Purified Fur protein was confirmed by Western blot assay with antibody provided by Dr. Michael Vasil.

#### 2.11.3 EMSA

EMSAs were carried out based on previously published protocols (de Lorenzo et al., 1988; Hassett et al., 1997). Specifically, the binding reaction was prepared as mixing the radio‐labeled DNA (<1 nmol L^−1^) with purified Fur protein (10 nmol L^−1^) in the binding buffer (10 mmol L^−1^ Bis‐Tris borate, 40 mmol L^−1^ KCl, 0.1 mmol L^−1^ MnSO_4_, and 1 mmol L^−1^ MgSO_4_, pH is 7.5) with 100 (μg ml^−1^) bovine serum albumin (Sigma) and 50 μg/ml poly dI‐dC (Thermo Scientific), followed by incubation at 37°C for 20 min. Where indicated, competitor DNA was added at a final concentration of 10 nmol L^−1^, 50 nmol L^−1^, and 100 nmol L^−1^, was added to the reaction mixture prior to the above incubation with Fur. Following this incubation, protein‐bound and unbound DNA fragments were separated by electrophoresis on an 8% polyacrylamide gel and radioactive signals detected using a phosphor‐imaging system.

### 2.12 Enzymatic RNA structure probing

#### 2.12.1 Construction of the run‐off plasmid pT7‐T

Complementary oligonucleotides containing the nucleic acid sequences of the T7 promoter, the full *shuT* 5′ UTR, and the first five codons of *shuT* (Table S2) were combined and annealed in STE buffer (0.1mol L^−1^ NaCl, 10 mmol L^−1^ Tris‐HCl, 1 mmol L^−1^ EDTA, pH 8.0) by boiling in a water bath for 10 min followed by slow cooling to room temperature. The generated double‐stranded DNA was then ligated into plasmid pXG10 (Urban & Vogel, 2007) that had been digested by restriction enzymes AatII (New England Biolabs inc.) and NheI (New England Biolabs inc.) to generate the run‐off plasmid pT7‐T, from which the 5′ portion of the *shuT* transcript would be generated for subsequent enzymatic RNA structure probing analysis.

#### 2.12.2 *In vitro* transcription

*E. coli* strain DH5α carrying the run‐off plasmid pT7‐T was cultured to the stationary phase of growth in 3 ml LB broth with 30 (μg ml^−1^) chloramphenicol at 37°C. Following extraction, purified pT7‐T was linearized by digestion with restriction enzyme NheI‐HF (New England Biolabs Inc.) at 37°C for 2 hr. After purification by gel extraction, the linear DNA was used as template in an in vitro transcription reaction carried out according to the protocol provided by AmpliScribe^™^ T7‐Flash^™^ Transcription kit (epicenter, an Illumina company). Following completion of the in vitro transcription reaction, 1 μl of DNase was added to the reaction mixture, and the mixture was incubated at 37°C for 15 min. Next, RNA products were precipitated at −80°C for more than 15 min following addition of 1 ml 100% ethanol and 40 μl 3 mol L^−1^ sodium acetate (pH 5.2). Precipitated RNA was pelleted by centrifugation at 17,000 ***g*** for 15 min at 4°C and the RNA pellet washed twice by 1 ml ice‐chilled 70% ethanol followed by centrifugation as described above. After drying by centrifugation in a vacufuge set on “Alcohol mode” (Eppendorf) for approximately 2 min, the RNA pellet was rehydrated in 15 μl DEPC‐treated double‐distilled water.

#### 2.12.3 Radio‐labeling and enzymatic probing

Following synthesis and purification of the *shuT* transcript by in vitro transcription, the sample was treated with Calf Intestinal Alkaline Phosphatase (CIP) (New England Biolabs inc.) for 1 hr at 37°C to remove the 5′ triphosphate from each RNA molecule. Next, the CIP was removed from sample by phenol extraction and the RNA precipitated and dried as described above. The RNA pellet was rehydrated in 7 μl nuclease‐free water and 5′ end radio‐labeled as follows: T4 polynucleotide kinase (New England Biolabs Inc.) was used to transfer the γ‐^32^P‐phosphate group from γ‐^32^P‐ATP to the RNA sample. Radio‐labeled transcripts were purified and extracted from 8% acrylamide gel (UreaGel‐8, National Diagnostics) with elution buffer (10 mmol L^−1^ ethylenediaminetetraacetic acid (EDTA), 0.5% SDS, and 0.1 mol L^−1^ sodium acetate, pH 5.6), followed by RNA precipitation. Finally, structure probing assay was conducted using RNase T1 (Ambion), according to the procedures described in previous studies (Waldminghaus, Heidrich, Brantl, & Narberhaus, 2007). RNase T1 is used at either a 5‐fold or 10‐fold dilution as indicated.

### 2.13 In silico analyses

Promoter prediction: The putative *shuT* promoter region was identified using Bprom (http://linux1.softberry.com/berry.phtml?topic=bprom&group=programs&subgroup=gfindb) (Solovyev & Salamov, 2011).

Binding site prediction: Bprom and Virtual Footprint (http://www.prodoric.de/vfp/vfp_promoter.php) were used to identify the putative Fur‐binding site within *shuT* (Münch et al., 2005; Solovyev & Salamov, 2011).

RNA structure prediction: The secondary structure of *shuT* 5′ UTR and its stabilizing energy under various temperatures were predicted using Mfold (http://unafold.rna.albany.edu/?q=mfold/RNA-Folding-Form2.3) (Zuker, 2003).

### 2.14 Statistical analysis

All experimental analyses were performed in biological triplicate. An *F*‐test was conducted to test whether the comparing groups have equal variances or not, followed by the corresponding two‐tailed Student's t tests to determine significance (*p* ≤ .05).

## 3 RESULTS

### 3.1 shuT expression is regulated in response to iron availability by the transcriptional regulator Fur

**Figure 1:**
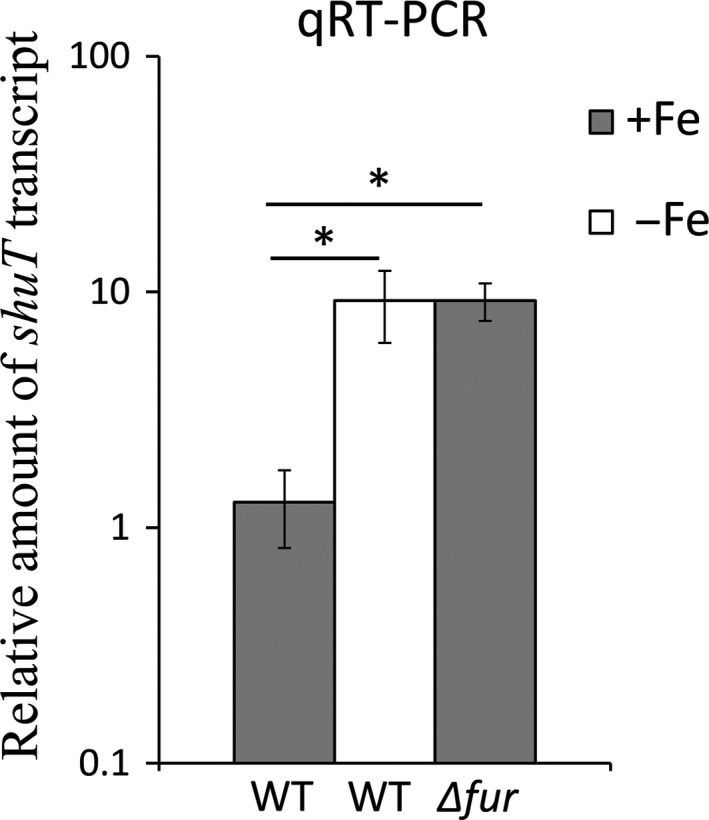
The expression of *shuT* is regulated in response to iron by the transcriptional regulator Fur. Quantitative real‐time PCR (qRT‐PCR) targeting *shuT* was performed following growth of wild‐type and *Δfur Shigella dysenteriae* to mid‐logarithmic phase at 37°C under the indicated culture conditions. “+Fe” indicates “iron‐rich” conditions achieved by culturing in Luria‐Bertani broth, whereas “−Fe” represents “iron‐poor” conditions achieved by culturing in Luria‐Bertani broth containing 150 μmol L^−1^ 2,2′‐bipyridine. Using the ΔΔCt method of calculation, the expression level of *shuT* is normalized to that of *rrsA* present in each sample and is expressed relative to the amount of the transcript present in the first wild‐type sample cultured under iron‐rich conditions. All analyses were carried out in biological triplicate. Error bars indicate the standard deviation and “*” indicates a statistically significance difference (*p*‐value ≤ .05)

To experimentally determine if expression of *shuT* is regulated in response to iron availability, quantitative real‐time PCR (qRT‐PCR) was performed to measure the relative amount of *shuT* transcript in wild‐type *S. dysenteriae* cultured to the midlogarithmic phase of growth under iron‐rich and iron‐poor conditions. Following growth under iron‐poor conditions, the relative amount of *shuT* transcript measured in wild‐type *S. dysenteriae* was significantly higher than that measured following growth of the strain under iron‐rich conditions (Figure 1). These data confirm that *shuT* expression is influenced by iron availability, and suggest that this regulation is mediated by an iron‐responsive alteration in transcriptional efficiency and/or transcript stability; an observation consistent with the prediction of Fur‐dependent regulation of *shuT* transcription. To test whether Fur is involved in mediating the observed iron‐dependent regulation of *shuT* expression, the relative amount of *shuT* transcript present in *S. dysenteriae* lacking *fur* was measured following growth of the strain under iron‐rich conditions, conditions under which Fur‐mediated repression would be predicted to occur. Deletion of *fur* eliminates the observed iron‐dependent decrease in the relative amount of *shuT* transcript, returning transcript levels to those observed in the wild‐type strain cultured under iron‐poor conditions (Figure 1). Taken together, these data demonstrate that the expression of *shuT* is regulated in response to iron availability and that the observed iron‐dependent decrease in *shuT* transcript levels is mediated, directly or indirectly, by the iron‐dependent transcriptional regulator Fur.

### 3.2 Identification of the transcriptional start site and promoter region of shuT

When direct, Fur regulates target gene transcription via iron‐dependent binding of the protein to specific DNA sequences, termed Fur‐binding site(s). For genes whose expression is directly repressed by Fur, the Fur‐binding site(s) most often overlap the promoter region of a target gene (Bagg & Neilands, 1987). In these cases, binding by the regulator effectively blocks binding by RNA polymerase, and by doing so, prevents transcription initiation.

**Figure 2:**
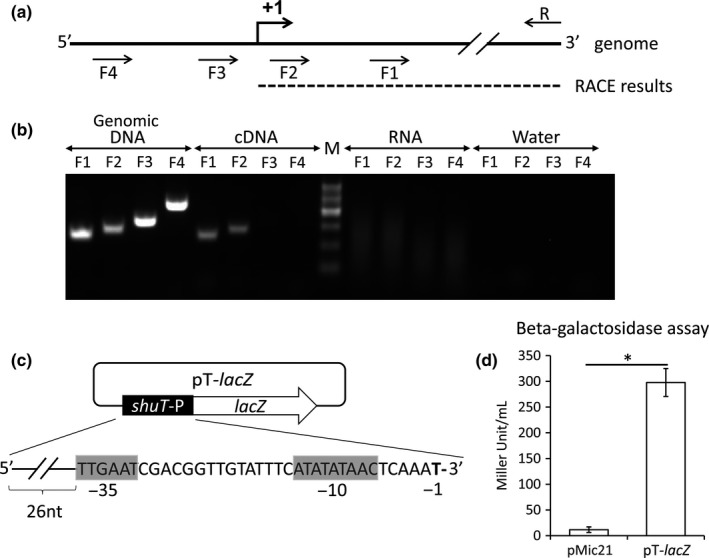
Identification of the transcription start site and promoter region of *shuT*. (a) A schematic of the *shuT* transcriptional start site obtained from 5′‐RACE analysis. The identified transcription start site is indicated with an arrow and “+1″. The solid line represents the genome of wild‐type *Shigella dysenteriae*, whereas the dashed line represents the furthest reading from 5′‐RACE analysis. Arrows represent the primers used in the reverse transcriptase PCR analysis shown in panel B. (b) Reverse transcriptase PCR confirmation of the existence of the *shuT* transcript and estimation of the length of the 5′ UTR. Total RNA was isolated from wild‐type *S. dysenteriae* cultured in LB broth containing 150 μmol L^−1^ 2,2′‐bipyridine and cDNA generated with random primers. Amplification was performed using the series of upstream primers (F1, F2, F3 & F4) and a single conserved downstream primer (R) depicted in panel A. Double‐ended arrows at the top of the image indicate the template used in each set of amplification reactions. Genomic DNA and water were used as positive and negative amplification controls, respectively. RNA template was used as template to demonstrate a lack of DNA contamination in each RNA sample. (c) A schematic of the plasmid pT*‐lacZ*. “shuT‐P” represents a 62‐nucleotide region of the genome of *S. dysenteria*e that starts from 26 nucleotides upstream of the putative promoter of *shuT* and extends to the nucleotide before the identified transcription start site. The −35 and −10 promoter regions are highlighted by gray boxes and the nucleotide before the predicted transcription start site of *shuT* is labeled as “−1″. (d) Measurement of beta‐galactosidase activity from wild‐type *S. dysenteriae* containing pT‐*lacZ* or the negative control plasmid pMic21. Plasmid pMic21 contains a promoter‐less *lacZ*, and function as a negative control; whereas plasmid pT*‐lacZ* has the putative promoter of *shuT* cloned immediately before the *lacZ* reporter gene. *S. dysenteriae* carrying the indicated plasmid was cultured to the midlogarithmic phase in LB broth with 150 μmol L^−1^ 2,2′‐bipyridine at 37°C. All analyses were carried out in biological triplicate. Error bars indicate one standard deviation and “*” indicates a statistically significance difference (*p*‐value ≤ .05)

The first required step in characterizing the molecular mechanism underlying Fur‐dependent regulation of *shuT* expression was to experimentally identify the transcription start site and promoter region of the gene. Based on the predicted transcript structure, four forward primers (F1, F2, F3, and F4) and one reverse primer (R) were generated and utilized in a series of reverse transcriptase PCR analyses designed to localize the *shuT* transcription start site (Figure 2a). The ability to amplify a specific product using each upstream primer (F1, F2, F3, or F4) paired with the conserved downstream primer (R) would indicate that the *shuT* transcript extends at least as far upstream as the binding site for the utilized upstream primer. While each primer set mediated amplification when *S. dysenteriae* genomic DNA was used as template, only primers F1 and F2 facilitated amplification when the amplification template was cDNA generated from RNA isolated from wild‐type *S.  dysenteriae* cultured under iron‐limiting conditions (Figure 2b). The isolated RNA was confirmed to be free of DNA contamination by the lack of amplification by any of the utilized primer pairs when the RNA itself was used as template (Figure 2b). Together these data demonstrate that the *shuT* transcription start site falls within the 28‐nucleotide region that separates primer F2 from primer F3.

**Figure 3:**
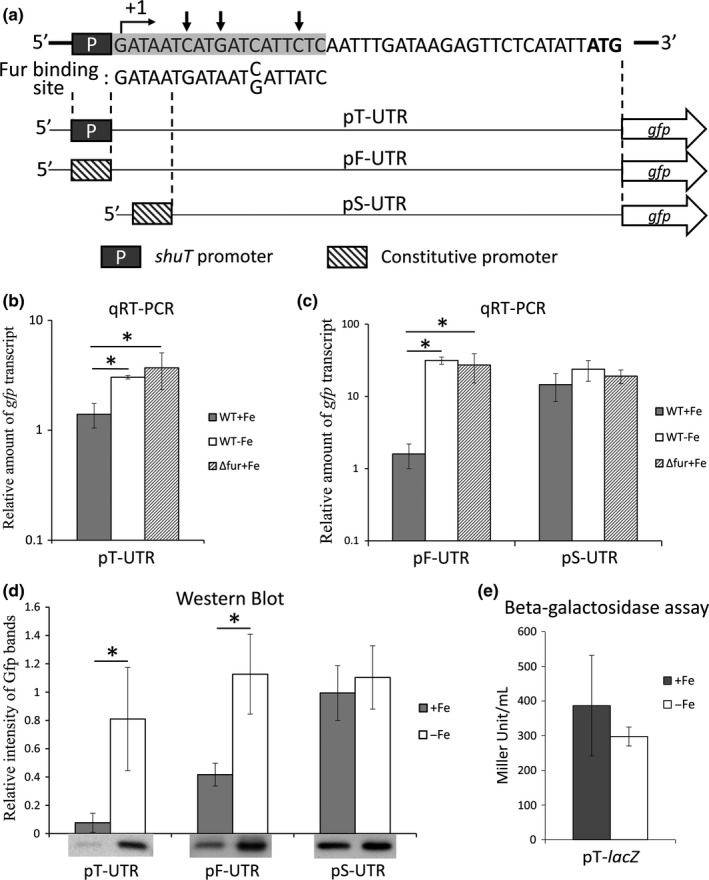
Iron‐dependent regulation requires a putative Fur‐binding site in the 5′ untranslated region of *shuT*. (a) Full sequence of the 5′ untranslated region of *shuT* as well as the sequence of a consensus Fur‐binding site. Nucleotides within the identified putative Fur‐binding site that differ from the consensus Fur‐binding site sequence are indicated with an arrow. Regions between the dashed‐lines indicate the region of *shuT* that has been inserted into the corresponding reporting plasmid (pT‐UTR, pf‐UTR, and pS‐UTR). (b and c) Quantitative real‐time PCR (qRT‐PCR) analyses of the relative levels of *gfp* transcript present in wild‐type and *ΔfurShigella dysenteriae* carrying the indicated reporter plasmid. Host cells were cultured under 37°C to midlogarithmic phase in either LB broth (+Fe for iron‐rich conditions) or LB broth containing 150 μmol L^−1^ 2.2′‐bipyridine (−Fe for iron‐poor conditions). Using the ΔΔCt method, the relative abundance of *gfp* is normalized to the amount of *rrsA* present in each sample and expressed relative to the amount of *gfp* transcript present in one of the wild‐type samples grown under iron‐rich conditions (WT+Fe). (d) Western blot analysis detecting Gfp levels in wild‐type *S. dysenteriae* carrying the indicated reporter plasmid following growth under the indicated conditions. Histograms indicate the relative intensity of the detected Gfp‐specific bands. (e) Measurement of beta‐galactosidase activity from wild‐type *S. dysenteriae* containing plasmid pT*‐lacZ*, a reporter plasmid on which the constitutive plasmid promoter immediately upstream of the *lacZ* reporter gene has been replaced with the *shuT* promoter. Cultures were grown to midlogarithmic phase in LB broth (+Fe) or LB broth containing 150 μmol L^−1^ 2.2′‐bipyridine (−Fe). All analyses shown in this figure were carried out in biological triplicate. Error bars indicate one standard deviation and “*” indicates a statistically significance difference (*p*‐value ≤ .05)

To further define the transcription start site of *shuT*, rapid amplification of cDNA 5′ end (5′‐RACE) analysis was performed using total RNA isolated from wild‐type *S. dysenteriae* grown to the midlogarithmic phase under iron‐poor conditions. Results obtained following sequencing of the products generated by 5′‐RACE are consistent with those obtained by reverse‐transcription PCR analysis and indicate that the transcription start site of *shuT* is located 42 nucleotides upstream of the start codon (Figures 2a,3a).

Guided by the experimentally confirmed location of the transcriptional start site and by in silico analysis (Bprom) a putative *shuT* promoter was identified (Solovyev & Salamov, 2011). The activity of the predicted *shuT* promoter was tested experimentally using beta‐galactosidase analyses. Specifically, a transcriptional reporter plasmid (pT*‐lacZ*) was constructed by cloning the predicted *shuT* promoter directly upstream of a promoter‐less *lacZ* gene. The cloned *S. dysenteriae* sequence contains only one predicted promoter, and ends with the nucleotide immediately 5′ to the identified transcription start site (Figure 2c). Wild‐type *S. dysenteriae* carrying reporter plasmid pT‐*lacZ* or the empty vector control (pMic21) were cultured to the midlogarithmic phase under iron‐poor conditions; and a basic beta‐galactosidase assay was used to determine if the sequence cloned into pT‐*lacZ* contains an active promoter. Significantly more beta‐galactosidase activity was measured from wild‐type *S. dysenteriae* carrying pT*‐lacZ* as compared to that of the strain carrying the promoter‐less negative control (pMic21) (Figure 2d). These data confirmed that the cloned sequences contain an active promoter, data consistent with the predicted localization of the *shuT* promoter (Figure 2c).

### 3.3 A functional Fur‐binding site is located immediately down‐stream of the shuT transcription start site

In order to experimentally determine if the observed iron‐dependent regulation of *shuT* expression is mediated by specific nucleic acid sequences within the 5′ UTR and/or promoter region of the gene, a transcriptional reporter plasmid (pT‐UTR) was constructed (Figure 3a). pT‐UTR carries an insert containing the native promoter and full 42‐nucleotide 5′ UTR of *shuT* cloned immediately upstream of a promoter‐less *gfp* reporter gene. Expression of *gfp* from the pT‐UTR reporter plasmid is under direct control of the identified *shuT* promoter and is subject to modulation by any regulatory element located within the promoter and/or 5′ UTR of the gene. Following introduction of pT‐UTR into wild‐type *S. dysenteriae* or *S.  dysenteriae Δfur*, production of Gfp protein and *gfp* transcript were measured via Western blot and qRT‐PCR analysis, respectively. The data obtained demonstrate that both the Gfp protein and transcript levels are significantly higher in wild‐type *S. dysenteriae* carrying pT‐UTR following growth of the strain in iron‐poor media as compared to the level of each following grown of the strain in iron‐rich media (Figure 3b,d). Furthermore, deletion of *fur* relieves the iron‐dependent inhibition of *gfp* transcription under iron‐rich conditions, as is seen upon analysis of *S. dysenteriae Δfur* carrying pT‐UTR (Figure 3b). Taken together, these data indicate that the cloned nucleic acid sequences, a fragment containing both the promoter and 5′ UTR of *shuT,* are sufficient to confer the observed iron‐dependent Fur‐mediated regulation.

In silico analyses using Bprom and Virtual Footprint were completed in order to identify and localize a putative Fur‐binding site within the sequences composing the *shuT* promoter and 5′ UTR. Using these approaches a 19‐nucleotide putative Fur‐binding site that differs by only three nucleotides from the consensus Fur‐binding site sequence was identified immediately downstream of the *shuT* transcription start site, from +1 to +19 of *shuT* (Figure 3a) (Lavrrar & McIntosh, 2003). The prediction of a Fur‐binding site within DNA encoding a 5′UTR is unusual, as in most cases of Fur‐mediated iron‐dependent repression, the Fur‐binding site overlaps with the −35 region of the regulated promoter.

To confirm that sequences within the *shuT* promoter region do not mediate iron‐dependent transcriptional regulation, a beta‐galactosidase assay using the reporter plasmid pT‐*lacZ* was conducted. This previously constructed reporter plasmid, pT‐*lacZ,* contains nucleic acid sequences composing just the *shuT* promoter region cloned immediately upstream of the promoter‐less *lacZ* reporter gene, sequences that do not include the putative Fur‐binding site (Figure 2c). As expected, no significant difference was measured in the amount of beta‐galactosidase activity detected from wild‐type *S. dysenteriae* carrying pT*‐lacZ* grown under iron‐rich or iron‐poor conditions (Figure 3e). These data confirm that the activity of *shuT* promoter itself is not influenced by iron availability.

To determine if nucleic acid sequences within the *shuT* 5′ UTR, the region containing the identified putative Fur‐binding site, are sufficient to confer iron‐dependent regulation, the reporter plasmid pF‐UTR was constructed. pF‐UTR contains the full 42‐nucleotide *shuT* 5′ UTR cloned between a constitutive plasmid promoter and the reporter gene *gfp* (Figure 3a). As cloned, expression of *gfp* from pF‐UTR is driven by the constitutive plasmid promoter and subject to regulation mediated solely via sequences contained within the cloned *shuT* 5′ UTR. Western blot and qRT‐PCR analyses were used to measure the relative amounts of Gfp protein and *gfp* transcript in wild‐type and *Δfur S. dysenteriae* carrying pF‐UTR, following growth of each strain under iron‐rich or iron‐poor conditions as indicated. The amount of Gfp protein (Figure 3d) and *gfp* transcript (Figure 3c) measured from wild‐type *S. dysenteriae* carrying pF‐UTR cultured under iron‐poor conditions were significantly higher than those measured following growth of the strain under iron‐rich conditions. Moreover, under iron‐rich conditions, the level of *gfp* transcripts produced by *Δfur* strain was significantly higher than that detected from wild‐type *S. dysenteriae* cultured under similar conditions (Figure 3c). Taken together, these data confirm that sequences within the *shuT* 5′ UTR are sufficient to confer iron‐dependent regulation and that this regulation is influenced, directly or indirectly, by the activity of Fur.

**Figure 4:**
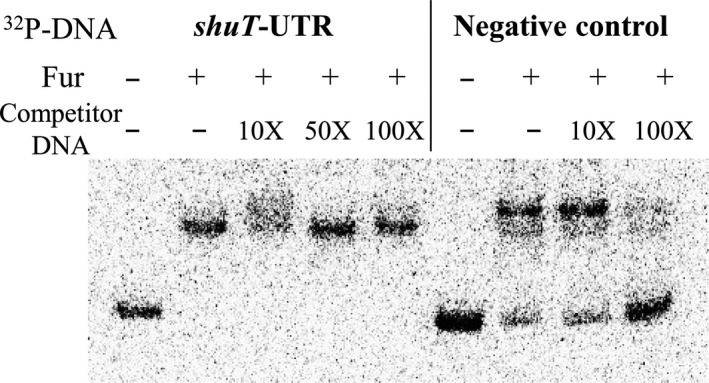
Fur binds specifically to sequences within the *shuT* 5′ UTR. Electrophoretic mobility shift assay testing direct binding between Fur and nucleic acid sequence within the *shuT* 5′ UTR. ^32^P‐labeled DNA fragments were generated by PCR amplification from plasmid pF‐UTR (*shuT‐*UTR) or pXG‐1 (Negative Control) using radio‐labeled primers. Fragment “*shuT*‐UTR”, which is amplified from pF‐UTR, contains the full 42‐nucleotide *shuT* 5′ UTR; whereas fragment “Plasmid”, amplified from pXG‐1, has the same sequence as “*shuT*‐UTR” but is lacking the 42‐nucleotide *shuT* 5′ UTR. For the left five reactions indicated by “shuT‐UTR”, nonlabeled “Negative Control” fragment serves as the competitor DNA; whereas for the right four reactions indicated as “Negative Control”, the competitor DNA is nonlabeled “*shuT*‐UTR”. The ratio between Fur protein (10 nmol L^−1^) and each radio‐labeled DNA species (<1 nmol L^−1^) is about 10:1; concentrations of the competitor DNA are either 10 times, 50 times, or 100 times higher than that of the labeled DNA as indicated. After incubating the binding reactions at 37°C for 15 min, samples were separated on an 8% acrylamide gel. “+” and “−” represents the existence of the indicated component

Following up on the finding that sequences encoding the *shuT* 5′ UTR harbor a putative Fur‐binding site and are sufficient to mediate iron‐dependent regulation, electrophoretic mobility shift assays (EMSA) were conducted to determine if Fur binds directly to these nucleic acid sequences. ^32^P end‐labeled primers were used to generate radio‐labeled DNA fragments via PCR amplification. Plasmid pF‐UTR, which has been confirmed to mediate iron‐dependent regulation, was used as PCR template to generate a DNA fragment containing the putative Fur‐binding site (Figure 3a). As a nonspecific control, an identical DNA fragment (negative control) lacking the *shuT*‐specific sequences was amplified from plasmid pXG‐1, which contains the constitutive promoter located immediately upstream of the *gfp* coding region. As shown in Figure 4, purified *Shigella* Fur protein binds both the DNA fragment containing sequences of the *shuT* 5′ UTR and the nonspecific control (Figure 4). Increasing amounts of competitor DNA (*shuT* 5′ UTR or nonspecific competitor) were added to the reaction mixture to determine specificity of binding between Fur and each DNA species. The addition of increasing amounts of unlabeled nonspecific control DNA did not disrupt binding between Fur and the *shuT* 5′ UTR. The addition of increasing amounts of unlabeled *shuT* 5′ UTR, on the other hand, competed Fur away from the labeled nonspecific DNA fragment (Figure 4). Taken together, these data indicate that Fur preferentially binds sequence within the *shuT* 5′ UTR; data supporting the conclusion that the observed Fur‐dependent regulation of *shuT* expression is direct.

If Fur‐dependent regulation of *shuT* is mediated by the identified putative Fur‐binding site, disruption of this site would be expected to decrease or eliminate the observed iron‐dependent regulation. To test this hypothesis directly, a transcriptional reporter plasmid (pS‐UTR) was constructed in which a truncated version of the *shuT* 5′ UTR was cloned between the constitutive plasmid promoter and the reporter gene *gfp* (Figure 3a). The truncated 5′ UTR cloned within pS‐UTR lacks the first five nucleotides of the predicted Fur‐binding site, deleting approximately one‐fourth of the putative site. Western blot and qRT‐PCR assays were carried out to measure the relative amounts of Gfp protein and *gfp* transcript produced by wild‐type *S. dysenteriae* carrying pS‐UTR following growth of the strain under iron‐rich and iron‐poor conditions. No significant differences in Gfp protein levels or relative amounts of *gfp* transcript were detected following growth of wild‐type *S. dysenteriae* carrying pS‐UTR under iron‐rich or iron‐poor conditions (Figure 3d). Additionally, the amount of *gfp* transcript measured in *Δfur S. dysenteriae* carrying pS‐UTR following growth in iron‐rich media was equivalent to that measured in wild‐type *S. dysenteriae* cultured in both iron‐rich and iron‐poor media (Figure 3c). Together these results demonstrate that, unlike sequences contained within the full‐length *shuT* 5′ UTR, sequences contained within the truncated *shuT* 5′ UTR do not confer iron‐dependent regulation. These data support the conclusion that the observed iron‐responsive, Fur‐dependent regulation of *shuT* expression is mediated by the Fur‐binding site identified within the 5′ UTR of the gene.

### 3.4 Nucleic acid sequences within the shuT promoter and/or 5′ UTR confer temperature‐dependent posttranscriptional regulation

**Figure 5:**
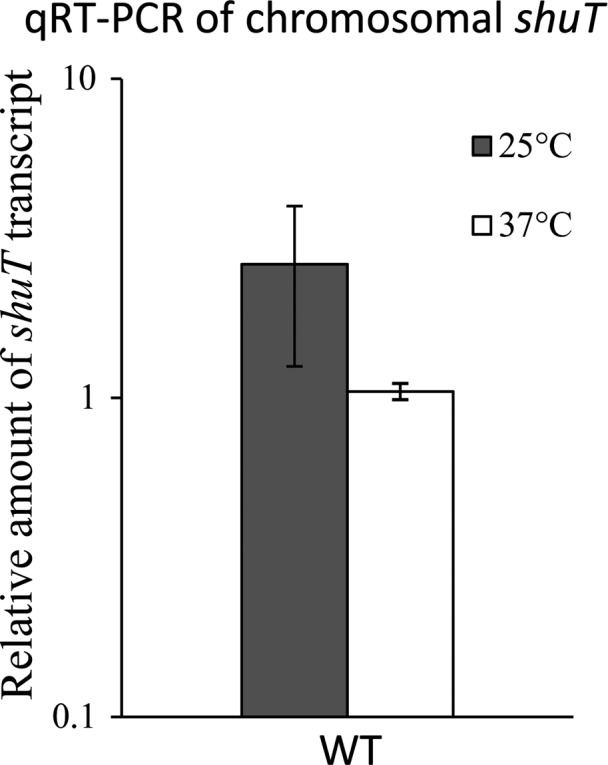
Environmental temperature does not influence the relative abundance of *shuT* transcript. The relative abundance of *shuT* transcript was measured using quantitative real‐time PCR in wild‐type *Shigella dysenteriae* following growth of the strain to the midlogarithmic phase under iron‐poor conditions at either 25 or 37°C. Iron‐poor conditions were achieved by culturing in Luria‐Bertani broth containing 150 μmol L^−1^ 2,2′‐bipyridine. Using the ΔΔCt method of calculation, *shuT* levels were normalized to that of *rrsA* present in each sample and are expressed relative to the amount of the transcript present in the first cultured at 37°C. All analyses were carried out in biological triplicate. Error bars indicate the standard deviation

In addition to iron‐limitation, environmental temperature also serves as a host‐associated environmental signal. Specifically, the increase in temperature from a variable room temperature of approximately 25°C to a constant 37°C corresponds to entry of a pathogen into the human body, and can trigger the production of factors that facilitate bacterial survival and/or virulence in this often harsh environment. Multiple virulence‐associated genes in *Shigella* are regulated in response to environmental temperature ((Schroeder & Hilbi, 2008). Previously, the gene encoding the outer‐membrane heme receptor of the Shu system, *shuA*, has been identified to be subject to temperature‐dependent posttranscriptional regulation via the activity of an RNA thermometer located within the 5′ UTR of the gene (Kouse et al., 2013). Whether temperature changes also influence expression of other *shu* genes, especially those encoded on transcripts separated from that of *shuA*, remained unknown. *shuT* is encoded in the opposite direction of *shuA*, with more than 300‐nucleotides separating the transcriptional start sites of these two genes (Wyckoff et al., 1998). While temperature does not influence the relative abundance of *shuT* transcript measured in wild‐type *S. dysenteriae* (Figure 5), in silico analysis predicts that the gene may be subject to posttranscriptional temperature‐dependent regulation. Specifically, in silico analysis using Mfold predicts the presence of a structure formed by the terminal 45 nucleotides (including the ATG) of the *shuT* 5′ UTR that, when formed, would occlude the SD site within the *shuT* transcript (Figure 7a) (Zuker, 2003). Moreover, the predicted stabilizing energy (ΔG) of this putative inhibitory structure becomes less negative as temperature increases, with a decrease in the absolute value of nearly twofold upon a temperature increase from 25 to 37°C (data not shown). The identification of a putative temperature‐sensitive structure within the *shuT* 5′ UTR that occludes the SD site supports the hypothesis that *shuT* translation is regulated in response to temperature via the activity of an RNA thermometer.

**Figure 6:**
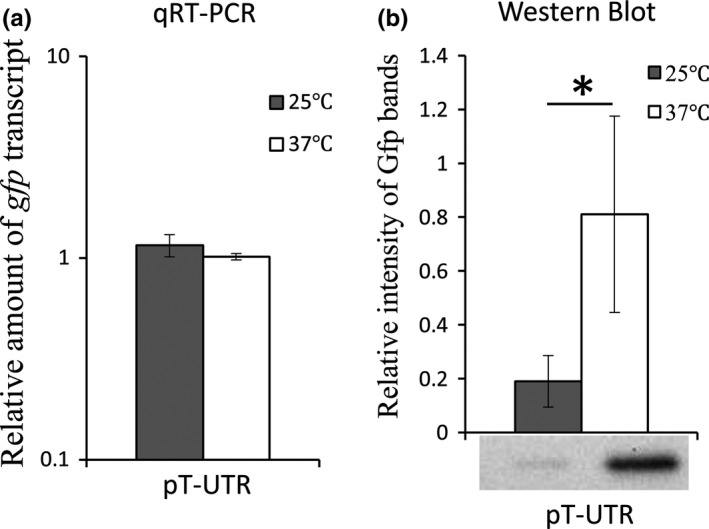
*shuT* is subject to temperature‐dependent posttranscriptional regulation. Wild‐type *Shigella dysenteriae* carrying pT‐UTR (reporter plasmid on which the constitutive plasmid promoter immediately upstream of *gfp* has been replaced by the *shuT* promoter and full‐length 5′ UTR) were cultured to midlogarithmic phase at 25 or 37°C in LB broth containing 150 μmol L^−1^ 2′‐ Bipyridine, and the relative amounts of *gfp* transcript or Gfp protein measured by quantitative real‐time PCR (qRT‐PCR) (a) or Western blot analysis (b), respectively. Protein and RNA samples were generated from the same set of cultures to ensure relevant comparisons. For the qRT‐PCR analyses, the ΔΔCt method was used to normalize the level of *gfp* to that of *rrsA* present in each sample and express it relative to that measured in the first 37°C sample. All analyses were carried out in biological triplicate. Error bars indicate one standard deviation and “*” indicates a statistically significance difference (*p*‐value ≤ .05)

To experimentally determine if nucleic acid sequences contained within the *shuT* promoter and 5′ UTR are sufficient to confer temperature‐dependent regulation, reporter plasmid pT‐UTR (Figure 3a) was used to determine if changes in environmental temperature result in altered expression of the reporter gene *gfp*. As described above, pT‐UTR carries the *shuT* promoter and full 5′ UTR region cloned immediately upstream of the start codon of a *gfp* gene. As a result of this arrangement, expression of *gfp* from the pT‐UTR is driven by the *shuT* promoter and subject to regulation via any regulatory element located within the *shuT* promoter and/or 5′ UTR. Following growth of wild‐type *S. dysenteriae* carrying pT‐UTR in iron‐poor media at either 25 or 37°C, the relative levels of both *gfp* transcript and Gfp protein were measured by qRT‐PCR and Western Blot analyses, respectively. Given that sequences within the 5′ UTR of *shuT* confer iron‐dependent regulation, iron‐poor media was used in these analyses to minimize the iron‐dependent repression of *shuT* transcription. The data obtained demonstrate that while there was no significant difference in the relative amount of *gfp* transcript measured following growth of the strain at either temperature tested (Figure 6a), Gfp protein levels were significantly higher following growth of the strain at 37°C as compared to those measured following growth of the strain at 25°C (Figure 6b). Taken together, these data demonstrate that the nucleic acid sequences within the *shuT* promoter and/or 5′ UTR are sufficient to confer temperature‐dependent posttranscriptional regulation onto expression of the reporter *gfp* gene; data that is consistent with regulation by an RNA thermometer.

### 3.5 Nucleic acid sequences composing the putative shuT RNA thermometer are sufficient to confer temperature‐dependent posttranscriptional regulation

**Figure 7:**
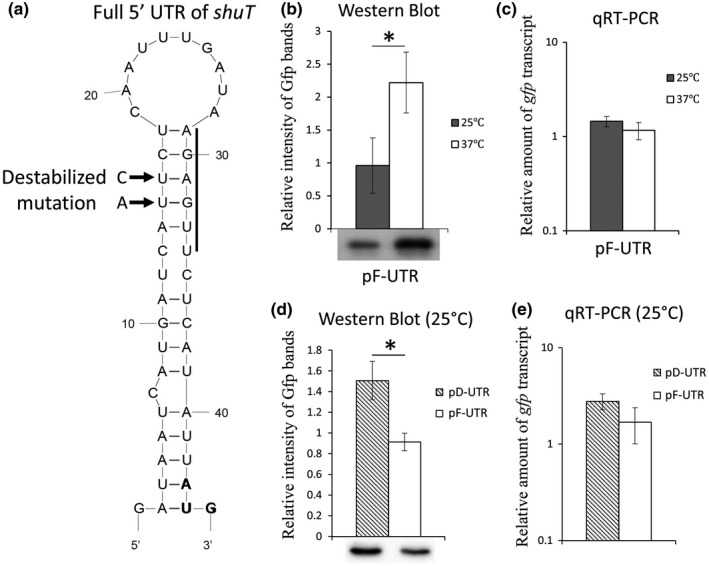
The 5′ untranslated region of *shuT* contains a functional RNA thermometer that is sufficient to confer temperature‐dependent posttranscriptional regulation. (a) A schematic of the secondary structure formed by the nucleic acid sequence of the *Shigella dysenteriae shuT* 5′ UTR as predicted by Mfold analysis. Sequences composing the ribosomal‐binding site are indicated by a bolded line and the translational start codon is in bold text. Arrows indicate the bases that were changed by site‐directed mutagenesis to generate the destabilized version of the element (U15 was changed to A and U16 was changed to C). (b and d) Western blot analysis detecting the relative amount of Gfp in wild‐type *S. dysenteriae* carrying the indicated reporter plasmid and grown under the indicated conditions. Histograms indicate the relative intensity of Gfp‐specific bands. (c and e) Quantitative Real‐time PCR (qRT‐PCR) analyzing the relative amounts of *gfp* transcript present in wild‐type *S. dysenteriae* carrying the indicated reporter plasmid and grown under the indicated conditions. Using the ΔΔCt method, the relative amount of *gfp* is normalized to the level of *rrsA* present in each sample and is expressed relative to the level measured in the first 37°C sample (c) or first pD‐UTR samples (e). In all analyses presented, the indicated bacterial strain was cultured to mid‐log phase in LB broth containing 150 μmol L^−1^ 2.2′‐bipyridine. Plasmid pF‐UTR contains a constitutive promoter and the wild‐type sequence of the full *shuT* 5′ UTR, followed by the reporter gene *gfp*; whereas plasmid pD‐UTR has the mutated 5′ UTR of *shuT* inserted between the constitutive promoter and the *gfp* reporter gene. Protein and RNA samples were generated from the same set of cultures to ensure relevant comparisons. All analyses were carried out in biological triplicate. Error bars indicate one standard deviation and “*” indicates a statistically significance difference (*p*‐value ≤ .05)

According to the in silico prediction, the 5′ UTR of *shuT* forms a single hairpin that resembles an RNA thermometer, a regulatory element that would be predicted to confer temperature‐dependent posttranscriptional regulation. To determine whether the observed temperature‐dependent regulation of *gfp* expression from pT‐UTR is mediated by nucleic acid sequences contained solely within the *shuT* 5′ UTR, wild‐type *S. dysenteriae* carrying the pF‐UTR reporter plasmid (Figure 3a) were grown in iron‐poor media at either 25°C or 37°C. As detailed above, pF‐UTR contains the full 42‐nucleotide *shuT* 5′ UTR cloned between a constitutive plasmid promoter and the reporter gene *gfp* (Figure 3a). As cloned, expression of *gfp* from pF‐UTR is driven by the constitutive plasmid promoter and subject to regulation mediated solely via sequences contained within the cloned *shuT* 5′ UTR. Following growth to midlogarithmic phase at 25 or 37°C, the relative amounts of *gfp* transcript and Gfp protein were measured by qRT‐PCR and Western blot analyses, respectively. The data demonstrate that Gfp protein levels were significantly higher following growth of the strain at 37°C as compared to those measured following growth of the strain at 25°C (Figure 7b), whereas *gfp* transcript levels shown no significantly difference (Figure 7c). Taken together, these data demonstrate that nucleic acid sequences within the *shuT* 5′ UTR, sequences that are predicted to form an RNA thermometer, are sufficient to confer temperature‐dependent posttranscriptional regulation.

### 3.6 A functional RNA thermometer is contained within the shuT 5′ UTR

Site‐specific mutagenesis was utilized to determine if the putative regulatory element identified within the *shuT* 5′ UTR is a functional RNA thermometer. Given that the regulatory activity of an RNA thermometer is dependent on a dynamic secondary structure, destabilizing the inhibitory hairpin structure of a functional RNA thermometer would be expected to alter its regulatory activity, specifically resulting in increased expression of the regulated gene at previously inhibitory temperatures. To destabilize the inhibitory hairpin within the *shuT* 5′ UTR, the uracils at position +15 and +16 (transcription start site is +1) were replaced by an adenine and a cytosine, respectively (Figure 7a). As was done for the construction of the wild‐type reporter (pF‐UTR), the mutated 5′ UTR of *shuT* was cloned between a constitutive promoter and the reporter gene *gfp*, making plasmid pD‐UTR (Figures 3a,7a). Since plasmid pD‐UTR and pF‐UTR are identical, with the exception of the destabilizing mutations within the cloned s*huT* 5*′* UTR, plasmid pF‐UTR was used as the wild‐type control in these analyses. According to the results of Western blot and qRT‐PCR analyses, *S.  dysenteriae* carrying pD‐UTR produced significantly higher amount of Gfp protein as compared to the strain carrying the wild‐type reporter pF‐UTR (Figure 7d) when each strain was cultured at the previously inhibitory temperature of 25°C; under these conditions *gfp* transcripts levels were equivalent (Figure 7e). Thus, as expected, destabilization of the inhibitory structures within the putative regulatory element results in more efficient translation of the target gene at a nonpermissive temperature. These data demonstrate that sequences within the *shuT* 5′ UTR of *shuT* contain a functional RNA thermometer.

### 3.7 Double‐stranded structure containing the shuT ribosomal‐binding site gradually opens when environmental temperature increases

**Figure 8:**
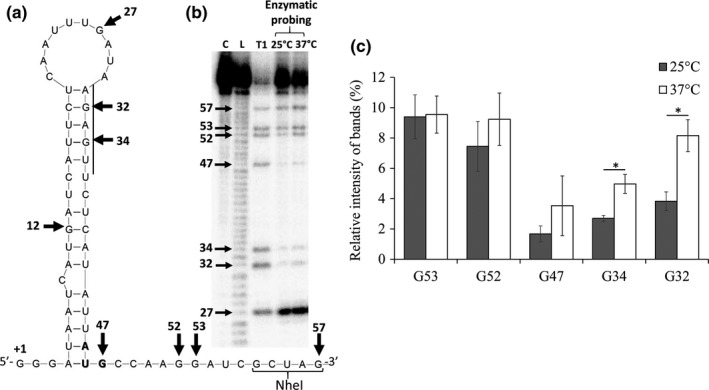
The inhibitory hairpin within the *shuT* RNA thermometer gradually opens as environmental temperature increases. In vitro transcribed RNA molecules were radio‐labeled at the 5′ end, and then partially digested by RNase T1, which specifically cut immediately 3′ to single‐stranded guanines. (a) Schematic of the *shuT* RNA thermometer with the Shine‐Dalgarno (SD) sequence highlighted with a line, the start codon in bold text and sequence from the engineered NheI site bracketed. All potential RNase T1 cutting sites are indicated with arrows. (b) Representative gel showing the radio‐labeled bands generated by digestion of the in vitro transcribed *shuT* RNA molecules. Control lanes: lane C contains RNA samples prior to the enzymatic or alkaline digestion, showing the background digestion of the experiment processes, lane L contains the sequencing ladder, and lane T1 contains the bands generated by RNase T1 digestion of the denatured template. Experimental lanes: lanes labeled 25 and 37°C contain the bands resulting from digestion of the *shuT* RNA thermometer at the indicated temperature with RNase T1 at a 5‐fold dilution. (c) As a means to increase the sensitivity of the assay, it was repeated using a lesser concentration of RNase (10‐fold diluted) and the results quantified. Relative intensities of each band were normalized to that of the G27‐associated band in the same lane. G27 is predicted to be single‐stranded and thus subject to equal digestion at each temperature tested. All statistics were generated from three independent repeats. Error bars indicate one standard deviation. “*” indicates a statistically significance difference (*p*‐value ≤ .05)

A feature that defines an RNA thermometer is a temperature‐responsive alteration in structure that results in increased access of the ribosomal‐binding site at permissive temperatures. To determine if the secondary structure of the *shuT* RNA thermometer truly responds to changes in environmental temperature, enzymatic probing of the in vitro transcribed *shuT* RNA thermometer was performed at experimentally validated permissive (37°C) and nonpermissive (25°C) temperatures using RNase T1, an RNase that specifically digests 3′ of each single‐stranded guanine (Figure 8a,b). In order to account for the potential difference of enzyme activity at different temperatures, the intensity of each detected band resulting from digestion at each native guanine is expressed relative to that of the band resulting from digestion at G27, a site that is predicted and confirmed by this experiment to be single‐stranded at each temperature tested (Figure 8a,c). Completion of the structure probing analyses revealed that as the temperature increased from 25 to 37°C, the band intensity associated with G32 and G34 increased significantly, indicating increased sensitive of these residues to digestion by RNase T1 (Figure 8c). These results indicate that the double‐stranded structure containing the ribosomal‐binding site of *shuT* gradually opens when the environmental temperature increases. Moreover, the relative intensity of the band resulting from digestion at G32 has a higher fold change (about 2.1‐fold) when the temperature increases than that associated with digestion at G34 (about 1.8‐fold), data suggesting that the dissociation of base pairs initiates from the loop region and proceeds down the length of the hairpin stem. Aside from those associated with digestion at position 32 and 34, none of the detected bands resulting from digestion at other native guanine residues demonstrated a significant change in intensity at the temperatures tested, data suggesting that these regions of the structure are less sensitive to alterations in environmental temperature (Figure 8c). Together these data demonstrate that the region of the *shuT* RNA thermometer structure containing G32 and G34 is most sensitive to alterations in environmental temperature, a sensitivity that would preferentially expose the SD sequences at permissive temperatures.

### 3.8 Heme utilization in S. dysenteriae is regulated by environmental temperature

**Figure 9:**
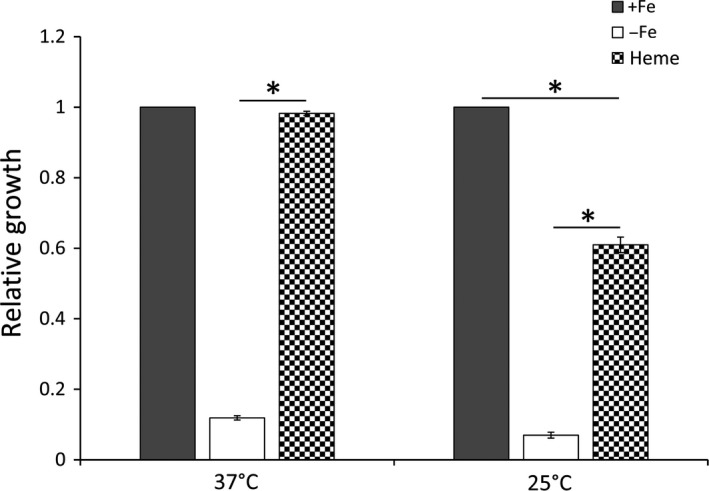
Heme utilization in *Shigella dysenteriae* is more efficient at higher environmental temperature. Wild‐type *S. dysenteriae* cells were grown to stationary phase under indicated conditions, and the optical density (OD_600_) was measured as an indication of growth. Values were normalized to that of cells cultured in LB broth (+Fe) at each temperature (37 or 25°C). Iron‐rich (+Fe) indicates growth of the strain in LB broth, whereas iron‐poor (−Fe) indicates growth in LB broth supplemented with 225 μmol L^−1^ 2,2′‐bipyridine. To measure growth in the presence of heme as the major source of iron (Heme) 100 (μg ml^−1^) heme was added to iron‐poor media. All analyses were carried out in biological triplicate. Error bars indicate one standard deviation and “*” indicates a statistically significance difference (*p*‐value ≤ .05)

Together with previous published data, two of the essential components of the Shu system have been characterized to having an RNA thermometer within the 5′ UTR of their transcript and subject to temperature‐dependent regulation. Specifically, these two components is outer membrane heme‐binding protein ShuA (Kouse et al., 2013) and periplasmic heme‐binding protein ShuT (this study). As a means to determine if the observed regulation translates into a physiologically relevant phenotype, a series of growth analyses were completed. Specifically, it was investigated whether temperature‐dependent regulation of *shu* genes translates into an impact on the ability of the pathogen to utilize heme as a sole source of iron at various temperatures. To this end, wild‐type *S. dysenteriae* was cultured to stationary phase under conditions with varied iron availability and the OD_600_ value measured as an indication of growth. LB broth alone was used as the iron‐rich (+Fe) condition, whereas iron‐poor (−Fe) condition was generated by adding 2,2′‐bipyridine to the LB broth to a concentration that inhibits the growth of wild‐type *S. dysenteriae* by about 90%. Heme was added to the iron‐poor (−Fe) media, generating a growth environment (Heme) in which heme represents the major source of nutritional iron. When cultured at 37°C, the addition of heme to the iron‐poor media restored growth of wild‐type *S. dysenteriae* to that observe in the iron‐rich media. When cultured at 25°C, however, the addition of heme to the iron‐poor growth environment did not return growth of wild‐type *S. dysenteriae* to the level observed for this strain when cultured in an iron‐rich environment (Figure 9). These data demonstrate that the ability of *S. dysenteriae* to utilize heme as a sole source of nutritional iron is influenced by temperature, with increased efficiency observed during growth of the pathogen at the temperature within the human body, the only environment where *Shigella* will encounter heme as a potential source of nutrient iron.

## 4 DISCUSSION

**Figure 10:**
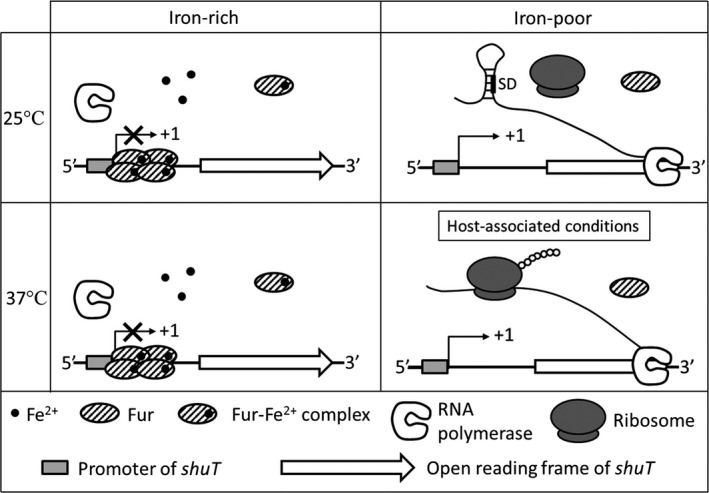
Model of *shuT* regulation in response to changes of iron availability and environmental temperature

The data presented in this study demonstrate that nucleic acid sequences harbored within the 5′ UTR of *S. dysenteriae shuT* confer both iron‐dependent transcriptional regulation and temperature‐dependent posttranscriptional regulation onto expression of the gene by distinct molecular mechanisms. Thus, as is the case of ShuA, ShuT production is limited to environments that mimic those encountered within the human host (low iron availability and relatively high temperature as compared to the nonhost environment), the only environment in which heme will be present as a potential source of nutritional iron (Figure 10) (Kouse et al., 2013; Mills & Payne, 1997). In addition, having multiple host‐associated environmental factors involved in regulating the production of multiple components of the heme acquisition system is likely beneficial to the pathogenic bacterium in at least two ways: (1) ensuring efficient response of the bacteria to changes of environmental factors; and (2) saving energy for other cellular processes when bio‐available iron sources are relatively abundant and/or when heme is not likely to be encountered. Furthermore, the coordinated regulation of multiple components of the *S. dysenteriae* Shu system likely increases fitness of the pathogen as these factors work in concert with one another and thus incomplete synthesis of the systems would provide limited benefit to the bacterium. Given that it is now established that both *shuA* and *shuT* are subject to posttranscriptional temperature‐dependent regulation, it remains a distinct possibility that the expression of the other *shu* genes is influenced in response to temperature, or another host‐associated environmental condition, by an as of yet unidentified regulatory mechanism(s) (Kouse et al., 2013).

The presented studies demonstrate that Fur mediates iron‐dependent transcriptional regulation of *shuT* expression by directly binding to nucleic acid sequences located immediately downstream of the transcription start site of the gene. The current model of the molecular mechanism underlying Fur‐mediated repression of bacterial gene expression is that binding of the Fur protein within the promoter region of the regulated gene prevents the initiation of transcription by physically blocking binding of RNA polymerase (Troxell & Hassan, 2013). For genes whose transcription is repressed by Fur, Fur‐binding sites are most commonly located adjacent to, or overlapping with, the −35 region of the gene's promoter (Christoffersen, Brickman, Hook‐Barnard, & McIntosh, 2001; Escolar, de Lorenzo, & Pérez‐Martín, 1997; Escolar, Pérez‐Martín, & de Lorenzo, 1998). Located immediately downstream of the *shuT* promoter, the Fur‐binding site mediating Fur‐dependent regulation of *shuT* expression is in a relatively unique location with respect to the *shuT* promoter. Although the precise molecular mechanism remains to be characterized, it is possible that, similar to the accepted model of Fur‐mediated regulation, binding of Fur to nucleic acid sequences immediately downstream of the promoter region acts to prevent transcription initiation by physically blocking binding by RNA polymerase. This regulatory model is supported by previous studies demonstrating that even with a core binding of only 19 nucleotides, in a DNA foot‐printing assay Fur binding protects no <31 nucleotides, and potentially up to 100 nucleotides, due to the fact that Fur can form multimeric complexes (Baichoo & Helmann, 2002; Escolar, Pérez‐Martín, & Lorenzo, 2000). A second potential mechanism underlying Fur‐mediated repression of *shuT* expression is that binding by Fur prevents transcription elongation by physically blocking the processivity of RNA polymerase. The possibility of such a mechanism is supported by the identification of functional Fur‐binding sites within the coding region of four genes in *E. coli*:* gspC*,* garP*,* yahA*, and *fadD* (Chen et al., 2007).

The *shuT* RNA thermometer is the second RNA thermometer located within the *S. dysenteriae shu* locus to be identified and characterized to date; the first being that housed within the 5′ UTR of *shuA* (Kouse et al., 2013). Although these two RNA thermometers are located in the same gene locus and regulate components of the same transportation system, they are distinct from each other in at least two important ways; distinctions that result in them being classified within two different families of RNA thermometers. First, the *shuT* 5′ UTR contains only 42 nucleotides and forms a single hairpin, a hairpin that functions as the *shuT* RNA thermometer. As a comparison, the *shuA* 5′ UTR is approximately 300‐nucleotide in length and is predicted to contain eight hairpins, the 3′‐most of which having been identified as the functional RNA thermometer (Kouse et al., 2013). The role of the remaining nucleic acid sequences and/or structures within the *shuA* 5′ UTR in controlling expression of the gene in response to temperature, or other signals, remains unknown. Second, a comparison of *shuT* and *shuA* reveals differences in the nucleic acid sequences of both the SD and anti‐SD regions of each. Significantly, unlike the *shuT* RNA thermometer, the *shuA* RNA thermometer contains four consecutive uracil residues that base pair with sequences of the SD region within the transcript to form the inhibitory structure of the element, a feature that results in its classification as a member of the FourU class of RNA thermometers (Kouse et al., 2013).

When the *shuT* RNA thermometer is distinct from that of *shuA*, it does share at least four similarities with the RNA thermometer shown to regulate expression of a small heat shock protein, Hsp17, in cyanobacteria *Synechocystis* (Kortmann, Sczodrok, Rinnenthal, Schwalbe, & Narberhaus, 2011). The features conserved between the *shuT* and *hsp17* RNA thermometers include: (1) a single stem‐loop structure formed by sequences constituting the entire 5′ UTR; (2) complete pairing between sequences of the SD site and anti‐SD site; (3) the presence of both an asymmetric internal loop and a large terminal loop within the inhibitory structure; and (4) the vast majority of the of the inhibitory structure being composed of relatively weak A‐U or G‐U pairs, with only two G‐C pairs predicted. These features in the *hsp17* RNA thermometer, particularly the presence of an asymmetric internal loop and the low number of G‐C pairs ensure the thermo‐sensing property of the hairpin by making it increasingly responsive to alterations in environmental temperature, a functional feature likely conserved in the *shuT* thermometer (Wagner, Rinnenthal, Narberhaus, & Schwalbe, 2015). Given the conservation of key features, *shuT* represents a second member of the now formed *hsp17*‐like family of RNA thermometers. The identification and characterization of additional members of this newly formed family of RNA thermometers will advance the foundational understanding of not only how these elements function, but also of what cellular processes they control. Such knowledge is essential to improving the ability to first identify RNA thermometers, and second manipulate their activity for research, industrial and/or therapeutic applications.

This study provides an example of two regulatory elements existing within the same sequence region, yet functioning to regulate gene expression via distinct molecular mechanisms. Such complex and multileveled regulation highlights the importance of the regulated processes to the survival and/or virulence of bacterial pathogens. Continuing studies to further elucidate the molecular events underlying known regulatory mechanisms, as well as those aimed at revealing novel regulatory mechanisms are critical to gaining a comprehensive understanding of bacterial pathogenesis. Such and understanding has the potential to advance the development of therapeutic agents to disrupt these key processes, and by doing so, limit the morbidity and mortality associated with bacterial infection.

## CONFLICT OF INTEREST

None declared.
