# Supplementary material for: Transcriptional and posttranscriptional regulation of Shigella shuT in response to host‐associated iron availability and temperature
Source: Microbiologyopen. 2017 Jan 26;6(3):e00442. doi: 10.1002/mbo3.442 (PMC5458455; doi:10.1002/mbo3.442)
Supplement: Supplementary file 1 [file MBO3-6-na-s001.pdf]

Supplementary table 1: Strains and plasmids used in this study.

Destination	Description	Reference/ source
**Strains**		
Escherichia coli		
DH5α		Life Technologies
Shigella dysenteriae		
O-4576S1-A (aka ND100)	Wild-type *S. dysenteriae*	(Murphy and Payne, 2007)
Δfur	*fur* deletion in O-4576S1-A	(Murphy and Payne, 2007)
**Plasmids**		
pHJW20	Cloning vector that has a *lacZ* gene, whose promoter region can be cut out by the restriction enzymes SalI and XbaI. Cm^r	(Castellanos *et al.*, 2009)
pT-*lacZ*	Reporter plasmid that has the *shuT* putative promoter region inserted right before the reporter gene *lacZ* of plasmid pHJW20. Cm^r	This study
pMic-21	pHJW20 carrying a Promoter-less *lacZ* gene, working as the negative control of β-galactosidase assay. Cm^r	(Castellanos *et al.*, 2009)
pXG10	Low-copy plasmid containing a PLtetO-1 constitutive promoter and a report gene *gfp*, which lacks the start codon. Cm^r	(Urban and Vogel, 2007)
pT-UTR	Reporter plasmid that has the *shuT* promoter, 5' UTR, and start codon inserted right before the reporter gene *gfp* of plasmid pXG10. Cm^r	This study
pF-UTR	Reporter plasmid that has the *shuT* full 5' UTR and start codon inserted right before the reporter gene *gfp* of plasmid pXG10. Cm^r	This study
pS-UTR	Reporter plasmid that has the truncated *shuT* 5' UTR and start codon inserted right before the reporter gene *gfp* of plasmid pXG10. Cm^r	This study
pD-UTR	Reporter plasmid that has the mutated *shuT* 5' UTR and start codon inserted right before the reporter gene *gfp* of plasmid pXG10. Cm^r	This study
pXG-1	Plasmid that have the PLtetO-1 constitutive promoter immediately in front of the coding region of gfp. Used in electrophoresis mobility shift assay to produce DNA fragment working as negative control. Cm^r	This study
pGEX-2T	Purchased plasmid vector containing a gene encoding glutathione S-transferase (GST), whose expression is controlled by promoter Ptac. In frame cloning sites exist after GST coding region. Amp^r	GE Healthcare

pGEX-*fur*	Plasmid that has the coding region of Shigella Fur cloned in frame after the coding region of GST. Used to produce the GST-Fur fusion protein, which is induced by IPTG. Amp^r	This study
pGEM-T easy vector	Purchased plasmid vector that has thymine overhang at the 3' end of each strand and can be used to ligate with PCR products directly. Amp^r	PROMEGA, Madison, MI
pT7-T	Cloning vector that has the T7 promoter leading the transcription of a 57-nucleotide transcript that includes the full 5' UTR and 5 codons of *shuT*, followed by a NheI cutting site. Cm^r	This study

Cm^r: resistant to chloramphenicol; Amp^r: resistant to ampicillin.

Supplementary table 2: Primers used in this study.

Primer	Sequence	Function
shuT-R	CAGTTTGGCGGTTTCTG	Reverse transcriptase PCR identifying the region of the *shuT* transcription start site
shuT-F1	CAAGGATCATCACTAGGC	
shuT-F2	TTCTCAATTTGATAAGAGTTCTC	
shuT-F3	TTGAATCGACGGTTGTATTTC	
shuT-F4	ATATCTCTGGGTTCTCAGC	
shuT-1-3	CTAGAATTTGAGTTATATATGAAATACAACCGTCGATTCAATACGCAAGGCGTTACAAGCGTATTTAG	Constructing plasmid pT-*lacZ* for β-galactosidase assay
shuT-1-5	TCGACTAAATACGCTTGTAACGCCTTGCGTATTGAATCGACGGTTGTATTTCATATATAACTCAAATT	
shuT-100	CCCCTTGTTCAGGAACCTGTAACG	Localizing the transcription start site by rapid amplification of cDNA 5' end
shuT outer	TTTGGCGGTTTCTGGTGGATAAGATGTC	
5'-RACE outer primer	GCTGATGGCGATGAATGAACACTG	
shuT inner	GTCAGCGATCCTCCTGCGACCACGATACG	
5'-RACE inner primer	CGCGGATCCGAACACTGCGTTTGCTGGCTTTGATG	
shuT-for-XG10-60	GTGACGTCTGCGTATTGAATCGACGG	Constructing plasmid pT-UTR
shuT-rev-XG10-2	GTGCTAGCCATAATATGAGAACTCTTATC	
shuT-5UTR-F	TGATAATCATGATCATTCTCAATTTGATAAGAGTTCTCATATTATGG	Constructing plasmid pF-UTR
shuT-5UTR-R	CTAGCCATAATATGAGAACTCTTATCAAATTGAGAATGATCATGATTATCATGCA	
T-RNAt F	TTCATGATCATTCTCAATTTGATAAGAGTTCTCATATTATGG	Constructing plasmid pS-UTR
T-RNAt R	CTAGCCATAATATGAGAACTCTTATCAAATTGAGAATGATCATGAATGCA	
shuT-2D-F	TGATAATCATGATCAACCTCAATTTGATAAGAGTTCTCATATTATGG	Constructing plasmid pD-UTR
shuT-2D-R	CTAGCCATAATATGAGAACTCTTATCAAATTGAGGTTGATCATGATTATCATGCA	
shuT-RNAT-F	CTAATACGACTCACTATAGGGATAATCATGATCATTCTCAATTTGATAAGAGTTCTCATATTATGCCAAGGATCG	Constructing plasmid pT7-T
shuT-RNAT-R	CGATCGCTAGGAACCGTATTATACTCTTGAGAATAGTTTAACTCTTACTAGTACTAATAGGGATATCACTCAGCATAATCTGCA	
Fur-gst tag-for	CGCGGATCCATGACTGATAACAATACCGCC	Constructing plasmid pGEX-*fur*
Fur-gst tag-rev	CCGGAATTCTTATTTGCCTTCGTGCG	

rrsAforRT	AACGTCAATGAGCAAAGGTATTAAC	Primers target *S. dysenteriae* house-keeping gene *rrsA* in qRT-PCR analyses
rrsArevRT	TACGGGAGGCAGCAGTGG	
GfpforRT	CCGTTCAACTAGCAGACCATTATC	Primers target reporter gene *gfp* in qRT-PCR analyses
GfprevRT	CTCATCCATGCCATGTGTAATCC	
pXG10-for	CTCTTACGTGCCGATCAACG	Primers used for colony screening and generating DNA fragments for gel mobility shift assay
pXG10-rev2	AGGTAGTTTTCCAGTAGTGC	
